# Virus Infection in Equine

**DOI:** 10.3390/ani12080957

**Published:** 2022-04-08

**Authors:** Amir Steinman, Oran Erster, Sharon Tirosh-Levy

**Affiliations:** 1 Koret School of Veterinary Medicine, The Robert H. Smith Faculty of Agriculture, Food and Environment, The Hebrew University of Jerusalem, Rehovot 7610001, Israel; sharontirosh@gmail.com; 2 Central Virology Laboratory, Ministry of Health, Haim Sheba Medical Center, Ramat-Gan 5265601, Israel; oran.erster@gmail.com

The relationship between men and horses has significantly evolved over the last century. Throughout history, horses have been used mainly for transportation, work, and war. In developing countries horses still serve as important burden animals for work and transport, while in more developed countries horses are being mainly used for sport, therapeutic riding or kept as pets. Additionally, in some countries, horses also serve as food animals for meat consumption. As a result, there is much interaction between horses and humans, with implications on human health as well as environmental consequences, under the One Health (OH) concept. Horses may serve as reservoirs to various pathogens, transmit certain zoonotic diseases and introduce pathogens into new geographical niches. The recent increase in human and animal international transportation, in combination with global warming and changing environment, has facilitated the spread of numerous infectious diseases into new geographic regions, as clearly demonstrated with the current SARS-CoV-2 outbreak.

One of the most well-known example for the introduction or re-introduction of pathogens to new niches is of West Nile virus (WNV). This mosquito-borne virus is maintained by wild birds and clinically effects mostly humans and horses [1]. Until the mid-1990’s, it was not considered an important differential diagnosis for equine and for human neurological disease and was mainly limited to Africa and the Middle East. During the 1990’s several large-scale outbreaks in humans and in horses were reported [2,3,4,5], including its dramatic appearance in the New York City area in 1999, followed by quick spread throughout the United States of America in the following years [6]. These events drew attention to the potential risk of introduction of pathogens into new global niches, further demonstrating the importance of better surveillance of equine viral diseases. WNV lineage 2 has been spreading with the detection of this lineage in European countries, first in Hungary in 2004 [7], and soon afterwards in neighboring countries [8]. In recent years, WNV lineage 2 has been detected in European countries including Spain in 2018 [9] and more recently in Germany [10]. In 2018, the largest outbreak of human WNV infections in Europe occurred, when 11 countries reported 1548 cases [11]. In the case of WNV, although it cannot be directly transmitted between horses and humans, horses can be used as sentinels for the activity of the virus in the area since they are mostly kept outside and are exposed to mosquitoes bites [1].

Another example for an emerging equine viral disease is Equine Encephalosis virus (EEV), which is reviewed in this special issue [12]. Until 2008, it was considered to be restricted to southern Africa. However, during 2008–2009, EEV was isolated in an outbreak in Israel, demonstrating the introduction of this pathogen into a new niche. In retrospective, this occurred even earlier since antibodies against EEV had already been detected in sera samples that were collected from horses in Israel in 2001 [13]. The spread of EEV from South Africa to central Africa [14], the Middle East [15], and India [16] is another example of the possible emergence of new pathogens in new niches and should be a reminder not to limit the differential diagnoses list when facing a possible outbreak, or a cluster of undiagnosed clinical cases.

Several other viruses have extended their geographical distribution in recent years, among which is Usutu virus (USUV). The apparent incidence of human USUV is very low compared with the incidence of WNV. However, the current knowledge might be biased by the low capacity to correctly identify USUV infection in humans [17]. In a study from Italy, in which cerebrospinal fluid and serum samples were retrospectively analyzed for the presence of USUV antibodies and RNA in human patients, it was concluded that USUV is not a sporadic event in the studied area [18]. In Israel, USUV was only isolated in six pools of mosquitoes in the north of country, five in 2015 and one in 2014 [19], however, until now, no human or equine clinical cases of USUV have been reported. In a survey that was conducted on equine sera samples in 2018, in Israel, 10.8% were seropositive [20], further supporting the possible role of horses as sentinels for the local circulation of such viruses, which might be otherwise underdiagnosed. 

Equine viral diseases can be divided into three groups: zoonotic pathogens, viruses with significant veterinary and economic significance, and viruses with minimal clinical impact on horses. 

Zoonotic viruses can be further divided to those that can be transmitted directly from horses to humans such as Rabies, Hendra and Venezuelan equine encephalitis (VEE) epizootic strains, and those that affect horses and humans but cannot be directly transmitted between them. These include WNV, USUV, Eastern Equine Encephalitis (EEE), Western Equine Encephalitis (WEE) and others. 

Rabies, is an example for a virus that can be directly transmitted from horses to humans, which is unfortunately still endemic in many countries, including Israel. Although horses can be infected, they are not an important source of human rabies infections. Yet, because of its severity, it is highly recommended to vaccinate horses in endemic areas, in order to prevent human infections. In Israel, between 1995 and 2019, rabies was detected in nine horses and in four donkeys [21]. No cases of rabies were detected in neither horses nor donkeys in 2020 [22]. During the same period (1995–2020), 1109 rabies cases were detected in other animals in Israel [22], further demonstrating the limited role of horses in the epidemiology of rabies. Although human cases are extremely rare in Israel, rabies vaccination is mandatory for competing horses and for imported horses and is highly recommended for all other horses.

Viruses that can cause severe economic losses include, for example, Equine Influenza virus (EIV) and Equine alpha Herpesvirus 1 (EHV-1). The economic impact of the EIV outbreak that occurred in Australia in 2007 was enormous, with an estimated financial cost to the governments of hundreds of millions of dollars, and the cost for the horse industry and associated businesses was even higher [23]. From an economic perspective, Equine Herpesviruses (EHV-1 in particular) are one of the most important equine pathogens in Europe [24]. EHV-1 may cause significant economic losses due to its high morbidity and the restrictions that are required to limit its spread. In three studies published in this special issue, the authors have evaluated the exposure of different cohorts to this important pathogen [25,26,27]. African Horse Sickness should also be included in this group and although it is still limited mainly to Africa, measures should be implemented to prevent its spread, which should include active surveillance as well as preventive vaccination in neighboring countries. 

The third group of viral pathogens include those that may cause mild to moderate disease to limited number of horses, and viruses that may not cause clinical signs and remain asymptomatic in horses. Several such viruses were recently found in horses, by either serology or by PCR, but their clinical significance is yet unknown. These include Equine Corona virus (ECoV) [28], Equine Parvoviruses [29], Equine Herpes virus-2, 5 [27] and Equine Encephalosis virus [12], which are described in this special issue. 

The overall aim of this Special Issue is to provide updated information on different aspects of equine viral diseases, including their prevalence, pathogenesis and diagnostics in different cohorts. 

El Brini et al. evaluated the seroprevalence of EHV-1 and EHV-4 among horses in the north of Morocco, as well as the antibody titers in vaccinated horses under field conditions. Overall, 12.8% unvaccinated and 21.8% vaccinated horses were positive to EHV-1 and all were positive to EHV-4 demonstrating that both viruses are endemic in the north of Morocco, with prevalence differences between regions. Furthermore, horses vaccinated with a monovalent EHV-1 vaccine had low antibody titers, which needs to be addressed [26]. 

El-Hage et al. aimed to better understand the role of EHVs infection in horses in Victoria, Australia, with and without clinical signs of respiratory disease, by PCR. Whereas only few horses were PCR positive for EHV-1 (three horses) and EHV-4 (five horses), many were PCR positive for EHV-2 (20.3%) and EHV-5 (60.2%). Although the odds of EHV-5 positive horses, demonstrating clinical signs of respiratory disease, were twice that of EHV-5 negative horses, no quantitative difference between mean loads of EHV shedding was found between the two groups and the clinical significance of respiratory gammaherpesvirus infections in horses is still unclear [27].

Bazanow et al. estimated the serological status of a semi-isolated group of horses (Huculs) in Poland, by using nasal secretions and sera samples. All the nasal swabs were negative for the tested viruses. Among the 20 horses that were tested, antibodies were detected against EHV-1 in 12 horses (60%), EIV A/H7N7 in 13 (65%), EIV A/H3N8 in 12 (60%), USUV in five (25%), and Equine Rhinitis A virus (ERAV) in one (5%), whereas antibodies against Equine Arteritis virus (EAV), Equine Infectious Anaemia virus (EIAV), and WNV were not detected. These results indicates that the Hucul herd could be used as sentinels for the detection of equine pathogens in the selected area [25]. 

Limited information is also available regarding Equine Parvoviruses, which were only recently identified. Pusterla et al. have tested the molecular prevalence of three Parvoviruses in blood and respiratory secretions of sick and healthy horses in the USA. Equine Parvoviruses were detected in both sick and healthy horses in similar frequencies suggesting that their role in equine respiratory disease is limited and should be further explored [29].

Schvartz et al. evaluated the risk of exposure to Equine Corona virus (ECoV) of horses in Israel. Exposure to ECoV was detected in 17 of 29 farms (58.6%) and in 41 horses (12.3%). The geographical area was the only factor that was found to be significantly associated with ECoV exposure. The results of this study indicate that ECoV should be included in the differential diagnosis list of pathogens in cases of adult horses with relevant clinical signs [28]. 

Lawton et al. have tested nasal secretions from equids with acute onset of fever and respiratory signs using qPCR and sera samples from healthy horses with possible exposure to humans with SARS-CoV-2 infection in the USA using ELISA. None of the clinical horses was found positive for SARS-CoV-2, whereas 35/587 (5.9%) apparently healthy Thoroughbred racing horses had detectable IgG antibodies to SARS-CoV-2. The authors have concluded that while horses appear to be susceptible to SARS-CoV-2 when in close contact with infected humans, they do not seem to be clinically affected [30].

Two reviews are included in this special issue, the first by Knox and Beddoe who reviewed current isothermal diagnostic techniques available for the detection of equine viruses of zoonotic concern, and provide insight into their potential for in-field deployment [31]. The second by Tirosh-Levy and Steinman who summarizes current knowledge regarding EEV structure, pathogenesis, clinical significance, and epidemiology [12].

Increased international transportation and trade over the last few decades have increased the risk of the introduction of pathogens into new areas. Global climate change has influenced environmental conditions and the ability of pathogens to survive, as well as changed the habitats of certain vectors and hosts. These processes have led to the emergence or re-emergence of various pathogens in different parts of the world, including those affecting horses. This special issue has featured some aspects regarding several well recognized as well as some new and emerging equine viral pathogens, highlighting the need of updated epidemiological data. Such surveillance is crucial for proper decision making by clinicians and by regulatory authorities. As well demonstrated by the recent global emergence of SARS-CoV-2, the development of an effective infrastructure for the rapid and effective detection and control of novel viral pathogens, as well as re-emerging ones is essential. Horses should play an important role in such surveillance system, not only for equine pathogens but also as sentinels to other viruses and arboviruses. As was demonstrated in several examples in this special issue, it is important to remember both as clinicians and as researchers, that when facing clinical cases, even when those are common, we should remain alert to the possibility of the intrusion of unknown pathogens and, therefore, should seek for a definitive diagnosis. This may allow for early detection of emerging or re-emerging pathogens. 

## Data Availability

Not applicable.
